# Treatment of High-Concentration Wastewater from an Oil and Gas Field via a Paired Sequencing Batch and Ceramic Membrane Reactor

**DOI:** 10.3390/ijerph17061953

**Published:** 2020-03-17

**Authors:** Yuan Wei, Yue Jin, Wenjie Zhang

**Affiliations:** 1 Guangxi Key Laboratory of Environmental Pollution Control Theory and Technology, College of Environmental Science and Engineering, Guilin University of Technology, Guilin 541004, China; wy0000000000@126.com; 2 College of Civil Engineering and Architecture, Guilin University of Technology, Guilin 541004, China; 2010053@glut.edu.cn

**Keywords:** oil and gas field, wastewater, biological treatment, functional microorganism, economic cost analysis

## Abstract

A sequencing batch reactor (SBR) and a ceramic membrane bioreactor (CMBR) were used in conjunction (SBR+CMBR) to treat high-concentration oil and gas field wastewater (HCOGW) from the China National Offshore Oil Corporation Zhanjiang Branch (Zhanjiang, Guangdong, China). The chemical oxygen demand (COD) and the oil concentrations in the wastewater were 20,000–76,000 and 600–2200 mg/L, respectively. After the SBR+CMBR process, the effluent COD and oil content values were less than 250 mg/L and 2 mg/L, respectively, which met the third level of the Integrated Wastewater Discharge Standards of China (GB8978-1996). Through microbiological analysis, it was found that the CMBR domesticated a previously unreported functional microorganism (JF922467.1) that successfully formed a new microbial ecosystem suitable for HCOGW treatment. In conjunction with the SBR process, the CMBR process effectively reduced pollutant concentrations in HCOGW. Moreover, economic analyses indicated that the total investment required to implement the proposed infrastructure would be approximately 671,776.61 USD, and the per-unit water treatment cost would be 1.04 USD/m^3^.

## 1. Introduction

The South China Sea is the deepest and largest sea in China and is extremely rich in oil and gas resources. Currently, many offshore oil drilling platforms operate in this region for subsea oil exploitation due to increasing oil demands. However, contaminants in petroleum wastewater are often very persistent and pose a threat to human and environmental health due to their known toxicity [1]. The South China Sea is an environmentally sensitive area, and the process of oil exploration and exploitation will have serious impacts on its marine ecology [2]. Thus, to protect the ecology of the South China Sea, on the mainland, the China National Offshore Oil Corporation (CNOOC) has built a treatment termination to collect large volumes of high-concentration oil and gas field wastewater (HCOGW) generated in the process of oil exploration and exploitation. However, HCOGW discharge without treatment will cause serious problems in the sewage treatment plant. Further treatment was needed to meet the third level of the Integrated Wastewater Discharge Standards of China (GB8978-1996).

Depending on the mining location, the chemical oxygen demand (COD) in the wastewater produced during offshore oil exploration and exploitation can reach up to 4730 mg/L [3], while BOD_5_ (i.e., the 5-day biochemical oxygen demand) values are often very low. Thus, compared with ordinary municipal sewage, it is much more difficult to treat offshore oil wastewater through biological processes. Physical and chemical methods commonly used in petroleum wastewater treatment include coagulation [4], electrochemical coagulation [5], and membrane technology [6,7]. However, the cost of chemical treatment of petroleum wastewater is too high [8]. Moreover, using membrane technology alone leads to membrane fouling and other problems [9,10], resulting in complex operation procedures and elevated maintenance costs [11]. Beyond that, physical and chemical treatments do not meet the concept of sustainable development. Compared with physical and chemical technology, biotechnology has become an important research alternative for industrial wastewater treatment due to its low cost and high efficiency. Biological processes have been successfully applied to the treatment of petroleum wastewater [12,13]. For instance, Schneider et al. [14] used a moving bed biofilm reactor (MBBR) for such purpose. After a 6-hour hydraulic retention time (HRT) and employing a bed-to-bioreactor volume ratio of 0.6, the effluent COD was 40–75 mg/L (i.e., which translated to a 69%–89% removal rate), and the NH_3_-N concentration was 2–6 mg/L (i.e., 45%–86% removal rate). Lang et al. [15] also treated petrochemical wastewater with *Sphingomonas*; after 48 hours, the total nitrogen (TN) removal rates were 94.22% and 90.10%, respectively.

Commissioned by CNOOC, the feasibility of biological methods treating HCOGW was evaluated in a lab-scale and pilot-scale experiments. Nevertheless, the BOD_5_ was very low; the results showed that cultivated sludge, including new strains, could treat HCOGW effectively. Following the results of the lab-scale and pilot-scale experiments, the full-scale sequence batch reactor (SBR) and the ceramic membrane reactor (CMBR) were designed and built to treat HCOGW. In this study, sludge from the Dongfang Municipal Sewage Treatment Plant (Hainan, China) was used as seed sludge. For the first time, the full-scale biological process was used for HCOGW treatment, and a SBR+CMBR process was practiced during this study. Flat-sheet ceramic membrane [16,17] and cultivated sludge were adopted in the CMBR process, and the new microbial community was expected to adapt the HCOGW. In this study, the treatment performance of the full-scale SBR+CMBR project was investigated. Above that, the functional microbial species in the process, as well as the infrastructure and operation costs, are discussed herein. 

## 2. Materials and Methods 

### 2.1. Treatment Process

**Figure 1:**
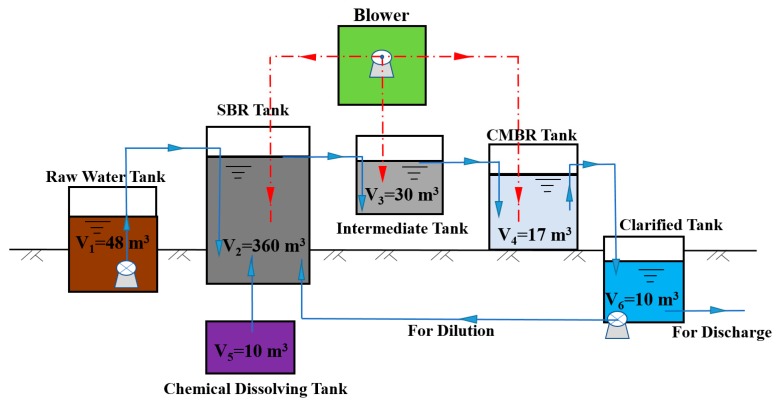
Process flow diagram.

The process flow is illustrated in Figure 1. The primary treatment system included a raw water tank and an SBR tank, which served the primary purpose of removing the easily degradable COD fraction. The secondary treatment system included an intermediate tank and CMBR tank. Its main purpose was to cultivate effective microorganisms, separate and biodegrade the remaining COD in wastewater.

As shown in Figure 1, HCOGW passes through the raw water tank, SBR tank, intermediate tank, CMBR tank, and clarified tank successively. Among them, the raw water tank is for regulating the quality and quantity of the HCOGW with an effective volume of 48 m^3^ and an HRT of 24 h. The SBR tank, intermediate tank, and CMBR tank are aerobic tanks, with effective volumes of 360 m^3^, 30 m^3,^ and 17 m^3^, respectively, and HRT of 24 h, 2 h, and 1 h, respectively. Part of the effluent flows back to the SBR tank to reduce the pollutant concentration in the influent. The reagent dissolving tank provides the necessary nitrogen, phosphorus, and other trace elements for the SBR tank. After entering the SBR tank, HCOGW was aerated for 24 h to degrade most of the COD. Then, HCOGW enters the intermediate tank to stabilize the water quality, and finally enters the CMBR tank to remove the remaining COD.

### 2.2. Analytical Methods

pH and temperature were measured with a Jenco 9010M pH meter (Jenco Instruments Inc., San Diego, CA, USA), and dissolved oxygen (DO) was measured with Jenco 6010M DO meter (Jenco Instruments Inc., San Diego, CA, USA). Oil content was measured by infrared spectroscopy (ET1200, Euro-Tech; Croydon, UK). Trans-membrane pressure (TMP) was recorded with a digital pressure sensor (SHANG YI; Foshan, China). Suspended solids (SS) and volatile suspended solids (VSS) in wastewater and sludge samples, as well as COD and BOD_5_, were determined according to Standard Methods [18,19,20]. The sludge settling ratio (SV30) was measured by the static sinking method of 100 mL measuring cylinder [18]. TN and total phosphorus (TP) were determined according to the method described by Yue et al. [20]. NH_3_-N was measured according to the method described by Zhang et al. [21].

### 2.3. Microbial Diversity Analysis

The methods are same as described by Zhang et al. [22].

## 3. Results and Discussion

### 3.1. Lab-Scale and Pilot-Scale Test

The quantity of HCOGW discharge was approximately 50 m^3^/d; wastewater quality parameters are summarized in Table 1.

HCOGW contains a large number of bio-refractory organic pollutants, resulting in the extremely high concentration of COD, ρ (BOD_5_) /ρ (COD) <0.02, and poor biodegradability. Generally, the COD of HCOGW is 20,000–30,000 mg/L. But during the overhaul of the plant area and drilling platform, a large number of organic matters in the pipeline and equipment will be flushed into the raw water tank, which will cause the COD in the raw water tank to rise sharply, and the maximum measured COD value is 76,000 mg/L. Given that HCOGW COD is very high, the treated wastewater was used to dilute in the SBR tank. In the late debugging period of engineering application, the influent COD concentration of the SBR tank was diluted below 3000 mg/L.

To ensure the successful application of the SBR+CMBR process in the full-scale application, we carried out lab-scale and pilot-scale experiments. In the lab-scale test stage, upflow anaerobic sludge blanket (UASB) and CMBR (UASB+CMBR) were used to treat HCOGW, and the treatment performance was confirmed. The effective volumes of the UASB reactor and CMBR reactor were 15 and 7.5 L, respectively, and the total HRT was 24 hours. The sludge from the anaerobic tank of the Yanshan Sewage Treatment Plant (Guilin, Guangxi, China) was used as the seed sludge to start the reactor. After dilution, the oil concentration of HCOGW was 600 mg/L, COD was about 2000 mg/L, and BOD_5_ was about 62 mg/L. The HCOGW was pumped into the UASB from the bottom of the reactor, and CMBR subsequently. During the stable running period, the effluent COD and oil concentrations treated by UASB+CMBR were 80–150 mg / L and 0.2–1.1 mg/L, which meet the third level of the Integrated Wastewater Discharge Standards of China (GB8978-1996). The results showed that biological processes could be used for HCOGW treatment.

In the pilot-scale test, as shown in Figure 1, the combination process of SBR + CMBR was adopted to treat HCOGW to ensure the feasibility of the engineering application and adjust the operational parameters. The results of the pilot-scale test showed that when the influent COD concentration was maintained at 5100 mg/L and the HRT was 5 days, and the total COD removal efficiency was above 97%, the total oil removal efficiency was as high as 99.6%, the effluent COD concentration was 100–200 mg/L, and the effluent oil concentration was less than 1 mg/L. 

### 3.2. Engineering Application

Based on the success of the lab-scale and pilot-scale test at the early stage, SBR+CMBR combined process was adopted in the full-scale process to treat HCOGW.

In the engineering application stage, the first step was the debugging stage, and it was the key period for the full-scale application. During this stage, the cycle of the SBR tank included two parts: the initial stage and the later stage. At the initial stage, it took 57 days, mainly including seed sludge feeding, screening of dominant bacteria, and sludge proliferation. At the later stage, it took 15 days, including system operation optimization, and sludge proliferation control. Because the pollutant load of the SBR tank was very high, it took a long time to complete sludge cultivation. CMBR tank was used to cultivate and separate the effective microorganisms. The effluent of the CMBR tank was designed to meet the discharge standards.

#### 3.2.1. Debugging Stage

**Figure 2:**
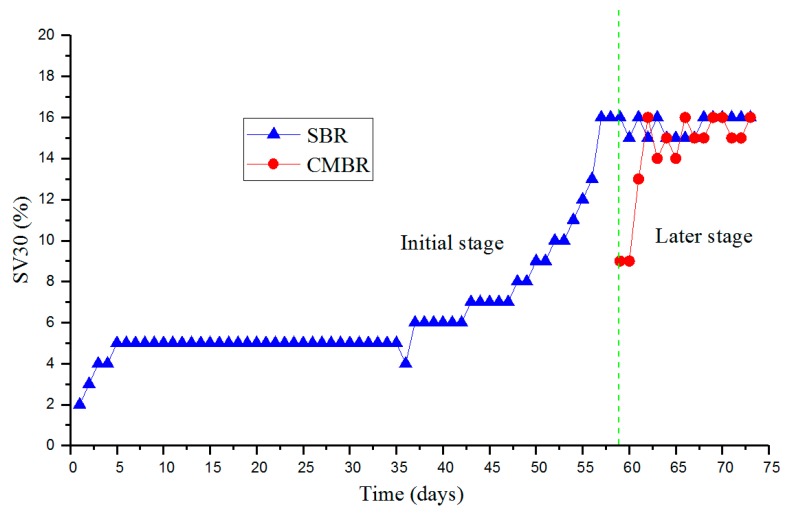
The sludge settling ratio (SV30) change during the debugging period.

The SV30 can be used as a reference for excess sludge discharge, and its value is generally 15%–30%. As illustrated in Figure 2, during the first 36 days, SV30 remained at approximately 5%. The SV30 then gradually increased from day 37 and reached the target value of 15% on day 57. In the later stage, the SV30 consistently fluctuated at approximately 15%, indicating that the startup of the SBR tank ended. In contrast, the SV30 in the CMBR tank stabilized at approximately 15% after roughly one week. Compared with the SBR tank, the target SV30 value in the CMBR tank was easier to achieve, which was attributed to the successful startup of the SBR tank. With low pollutant loads, the treatment performance of the CMBR tank was stable, which presumably enhanced the diversity and quantity of effective microorganisms capable of removing the remaining pollutants, thus facilitating an appropriate niche treating HCOGW. 

**Figure 3:**
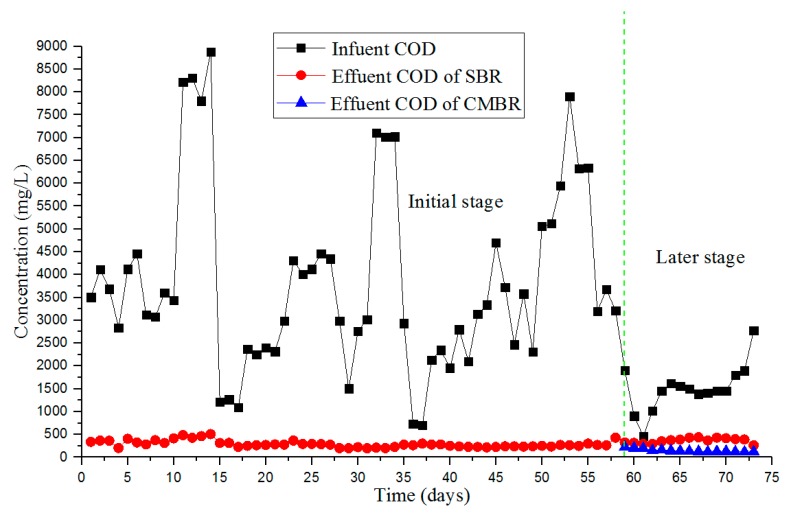
The chemical oxygen demand (COD) removal performance during the debugging period.

COD removal is illustrated in Figure 3. In the early debugging stage, the influent COD of the SBR tank was 700–8880 mg/L, while the effluent COD was 57–508 mg/L (Figure 3). Compared with the influent COD, the effluent COD value did not fluctuate substantially and remained at an average of 251 mg/L. In the later debugging stage, the effluent COD of the SBR tank remained stable at 262–440 mg/L with influent COD of 460–2780 mg/L, which indicated that the microorganisms in the SBR tank had adapted to the water quality of HCOGW and had formed a stable niche. At this point, the startup of the SBR tank was finished.

After CMBR, the effluent COD had substantially improved compared to the influent. Figure 3 illustrates that the effluent COD of the CMBR tank was 118–224 mg/L in the later debugging stage, and the average effluent COD value was 148 mg/L. The average effluent NH_3_-N concentration, TN concentration, TP concentration, oil concentration, and SS were below 15 mg/L, 60 mg/L, 3 mg/L, 0.5 mg/L, and 0 mg/L, respectively. During the debugging period, the effluent could meet the third level of the Integrated Wastewater Discharge Standards of China (GB8978-1996).

#### 3.2.2. TMP Changes

**Figure 4:**
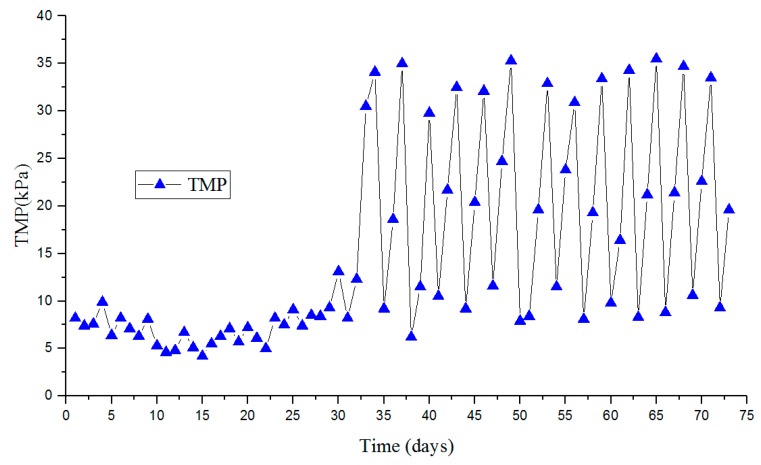
Trans-membrane pressure (TMP) changes during the study.

The adsorption and deposition of dissolved macromolecular organics, colloidal particles, and other materials in the mixed solution into the membrane pore lead to membrane fouling, thus increasing TMP [23,24,25]. As shown in Figure 4, when TMP was above 30 kPa, NaClO solution with a volume ratio of 0.1% was injected into the ceramic membrane by a pump to clean the inner part. During the cleaning process, the flow rate of NaClO solution was controlled at 0.4 L/min with 1 h. During the first 33 days, no chemical cleaning with NaClO solution was carried out due to the low TMP. During the debugging period, the inflow rate of CMBR was kept at a low value, so the TMP kept at a low level of 5-10 kPa with low membrane flux of below 15 L/(m^2^·h). From day 33, the membrane flux was increased to the designed value of 30 L/(m^2^·h). The TMP reached more than 30 kPa at this stage, chemical cleaning with NaClO solution was carried out every 4-5 days, and TMP was significantly reduced to about 10 kPa. The results showed that chemical cleaning with NaClO was effective in alleviating the membrane fouling in this study. Moreover, flat sheet ceramic membranes have a long lifetime and offer other advantages over polymer membranes for HCOGW treatment, such as simpler operation procedures and substantial cost reduction [12].

### 3.3. Microbial Diversity Analysis

**Figure 5:**
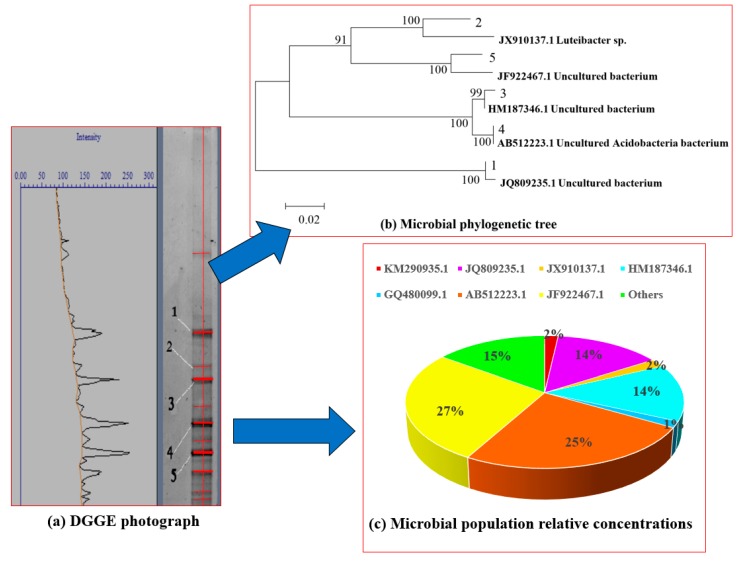
Microbial diversity analysis.

Denaturing gradient gel electrophoresis (DGGE) was carried out on CMBR sludge samples to characterize microbial populations. A DGGE map can distinguish microorganisms at the genus level; moreover, bright and faint bands in the gel can be interpreted as dominant and inferior microorganism populations, respectively [26]. As shown in Figure 5a, the dominant CMBR microorganism populations are represented by bands 4 and 5, with a total share of 52% (Figure 5c).

To further understand the relationship between bacteria in CMBR sludge, a phylogenetic tree was constructed. In the phylogeny tree, the horizontal branch represents the change in the evolution pedigree with time. The longer the branch length is, the greater the change in corresponding species of the branch is. In the vertical direction, we can see the evolutionary relationship between different species. The closer they are in the developing tree, the shorter the evolutionary divergence time of the species they represent, and the closer the genetic relationship is. As shown in Figure 5b, sequence JQ809235.1 matched bacteria from the phylum *Chlorobi* [27]. Furthermore, sequence JX910137.1 exhibited a 94% similarity with *Luteibacter sp.* characterized by Muangchinda et al. [28], and HM187346.1 had a 99% agreement with the *HDB-SIST494 16S RNA* gene characterized by Lin. [29] in deep oilfield water. Moreover, sequence AB512223.1 was found to belong to bacteria from the order *Burkholderiales* [30], and sequence JF922467.1 had a 96% similarity with the *B2-57 16S RNA* gene characterized by Liu et al. [31]. After gene comparisons, sequences JX910137.1 and JF922467.1 were confirmed to belong to new bacterial species (i.e., not previously reported). Additionally, KM290935.1 presented a 96% similarity with the *H7YL1YN01BV9QU 16S RNA* gene reported by Yang et al. [32].

Figure 5c illustrates the relative abundance of bacteria in the sludge sample. After 73 days of cultivation, the CMBR microbial population identified by sequence JF922467.1 became the most abundant, which suggested that this microbial population was largely responsible for the treatment of HCOGW in the CMBR tank. This contrasts with the observations of Jiang et al. [33] and Cappello et al. [34]. When these authors studied the treatment of refinery wastewater with an MBR, they determined that *Betaproteobacteria* were the main functional microorganisms. Due to different geological structures and mineral elements, the types and contents of pollutants in different geographical locations of offshore oil wastewater are also different. Therefore, when biochemical technology is used to treat petroleum wastewater in different regions, the types of main functional microorganisms are likely to be different. In this study, a new microbial community suitable for HCOGW treatment was developed by cultivating microorganisms in the CMBR process. The main functional microorganism (JF922467.1) has never been reported before.

### 3.4. Economic Evaluation

After the debugging period, the full-scale SBR+CMBR process was operated in a stable running period with a designed treatment capacity of 50 m^3^/d. We collected the running data of the full-scale SBR+CMBR process for one year. The treatment performance of the full-scale SBR+CMBR process during a stable running period is summarized in Table 2. Here, it can be observed that the SBR+CMBR process could effectively treat HCOGW with effluent COD of less than 250 mg/L and effluent SS of 0 mg/L. Beyond the requirements of the Integrated Wastewater Discharge Standards of China (GB8978-1996), the full-scale SBR+CMBR process showed a good NH_3_-N, TN, TP, and SS removal rate. Referring to the running data, we have carried out an economic evaluation of the full-scale SBR+CMBR process treating HCOGW. Total infrastructure investment and running costs are estimated in the following chapter.

#### 3.4.1. Total Infrastructure Investment

In an engineering application, the cost of total infrastructure investment is undoubtedly one of the important indexes to evaluate whether the treatment process is superior. To find out the economic benefits of the SBR+CMBR process in treating HCOGW, this study analyzed the investment and running cost of a full-scale SBR+CMBR process. All costs are based on the investment costs in the actual construction and operational process of the project.

The direct costs of implementing the proposed system include equipment, structure reinforced concrete, auxiliary building, and public works costs. Equipment costs included equipment investment and instrument costs, totaling 224,403.90 USD (the current exchange rate is 1 USD = 7.13 RMB). The system required the construction of a reinforced concrete structure with a 300 mm wall and tank bottom thickness costing 168.30 USD/m^3^. The total amount of concrete required for the wastewater treatment plant structure is V_1_ + V_2_ + V_3_ + V_4_ + V_5_ + V_6_ = 475 m^3^. Therefore, the total cost for reinforced concrete structures was 79,942.50 USD (i.e., 475 m^3^ × 168.30 USD/m^3^). The auxiliary facilities included the main building, blower room, machine repair, and power distribution room, garage, and warehouse, covering a total area of 670 m^2^. Assuming a building cost of 182.33 USD/m^2^ for this auxiliary unit, the total cost was 122,161.10 USD (i.e., 670 m^3^ × 182.33 USD/m^3^). Public works costs included plant road concrete, greening, and sewage treatment plant site purchase, totaling 97,711.08 USD.

Therefore, the direct costs were equal to the sum of the equipment, reinforced concrete structures, ancillary buildings, and public works costs, which amounted to 436,218.58 USD. Moreover, assuming that indirect costs were 30% of the direct costs (i.e., 130,865.57 USD), second part costs amounted to 10% of the direct costs (i.e., 43,621.86 USD), and project preparation costs were 10% of the sum of the direct, indirect, and second part costs (i.e., 61,070.60 USD), the total infrastructure investment (i.e., direct cost + indirect cost + second part cost + project preparation cost) was 671,776.61 USD.

**Figure 6:**
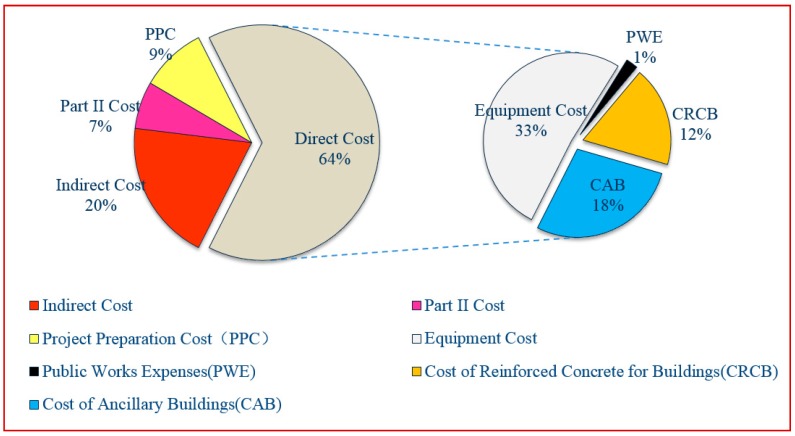
Total investment in infrastructure.

Figure 6 illustrates the total infrastructure investment distribution. Here, direct costs accounted for the vast majority of the total investment (i.e., up to 64%). Of these direct costs, equipment cost accounted for more than half of the total infrastructure investment.

#### 3.4.2. Running Cost

Power-associated costs mainly derived from the pump and blower operation, and the total daily power consumption was 87 kW. Assuming a 0.11 USD/kW cost, the annual power cost was 3493.05 USD. Due to the auto running mode of the plant, one worker with an average annual salary of 15,000.00 USD could operate and manage the plant; a 561.00 USD/year pharmaceutical fee was also contemplated. Considering that the approximate daily wastewater output was 50 m^3^/d, water treatment was 1.04 USD/m^3^ (i.e., total annual sewage treatment costs/wastewater output = (3493.05 USD + 15,000.00 USD + 561.00 USD)/(50 m^3^/d × 365 d)). During the running cost, employee wages accounted for 70.5% of the total sewage treatment cost.

## 4. Conclusions

Here, a full-scale SBR+CMBR process was designed and built to treat HCOGW. After treatment, the effluent quality could meet the third level of the Integrated Wastewater Discharge Standards of China (GB8978-1996). The SBR+CMBR process could cultivate and retain effective microorganisms in the tank; thus, a niche was formed to treat the HCOGW effectively. In the niche, new strains were determined to belong to unique functional microorganisms and domesticated in the CMBR tank during the study. The total investment required for the construction of the project was 671,776.61 USD, and the per-unit sewage treatment cost would be 1.04 $/m^3^.

## Figures and Tables

**Table 1:** Compositions of high-concentration oil and gas field wastewater (HCOGW).

Type	Parameter	Unit	Range	Average	Standard Deviation
Wastewater discharged from offshore oil rigs	Temperature	°C	18.3–22.4	--	--
	pH		6.5–9.1	--	--
	COD	mg/L	20,000—76,000	25,660	13,170
	BOD_5_	mg/L	36.1–650	338.5	237.3
	TP	mg/L	15–34.2	24.6	9.8
	TN	mg/L	1200–2736	2024	556
	Oil	mg/L	600–2200	1525	549

**Table 2:** Treatment performance of a full-scale sequencing batch reactor and a ceramic membrane bioreactor (SBR+CMBR) process during a stable running period.

Item	pH	COD (mg/L)	NH3-N (mg/L)	TN (mg/L)	TP (mg/L)	SS (mg/L)	Oils (mg/L)
HCOGW	6.5–9.1	30,000–40,000	650–820	1400–1620	22–25	500–600	600–2200
effluent	8.46–8.75	< 250	<30	<70	<5	0	<2
Integrated Wastewater Discharge Standards of China (GB8978-1996)	6–9	500	-	-	-	400	30
